# Monitoring the Control of Sexually Transmissible Infections and Blood-Borne Viruses: Protocol for the Australian Collaboration for Coordinated Enhanced Sentinel Surveillance (ACCESS)

**DOI:** 10.2196/11028

**Published:** 2018-11-20

**Authors:** Denton Callander, Clarissa Moreira, Carol El-Hayek, Jason Asselin, Caroline van Gemert, Lucy Watchirs Smith, Long Nguyen, Wayne Dimech, Douglas IR Boyle, Basil Donovan, Mark Stoové, Margaret Hellard, Rebecca Guy

**Affiliations:** 1 Kirby Institute University of New South Wales Sydney Sydney Australia; 2 Burnet Institute Melbourne Australia; 3 School of Public Health and Preventative Medicine Monash University Melbourne Australia; 4 National Reference Laboratory Melbourne Australia; 5 Research Information Technology Unit, Health and Biomedical Information Centre Department of General Practice University of Melbourne Melbourne Australia; 6 Sydney Sexual Health Centre Sydney Hospital Sydney Australia

**Keywords:** Australia, blood-borne viruses, public health, sentinel surveillance, sexually transmissible infections

## Abstract

**Background:**

New biomedical prevention interventions make the control or elimination of some blood-borne viruses (BBVs) and sexually transmissible infections (STIs) increasingly feasible. In response, the World Health Organization and governments around the world have established elimination targets and associated timelines. To monitor progress toward such targets, enhanced systems of data collection are required. This paper describes the Australian Collaboration for Coordinated Enhanced Sentinel Surveillance (ACCESS).

**Objective:**

This study aims to establish a national surveillance network designed to monitor public health outcomes and evaluate the impact of strategies aimed at controlling BBVs and STIs.

**Methods:**

ACCESS is a sentinel surveillance system comprising health services (sexual health clinics, general practice clinics, drug and alcohol services, community-led testing services, and hospital outpatient clinics) and pathology laboratories in each of Australia’s 8 states and territories. Scoping was undertaken in each jurisdiction to identify sites that provide a significant volume of testing or management of BBVs or STIs or to see populations with particular risks for these infections (“priority populations”). Nationally, we identified 115 health services and 24 pathology laboratories as relevant to BBVs or STIs; purposive sampling was undertaken. As of March 2018, we had recruited 92.0% (104/113) of health services and 71% (17/24) of laboratories among those identified as relevant to ACCESS. ACCESS is based on the regular and automated extraction of deidentified patient data using specialized software called GRHANITE, which creates an anonymous unique identifier from patient details. This identifier allows anonymous linkage between and within participating sites, creating a national cohort to facilitate epidemiological monitoring and the evaluation of clinical and public health interventions.

**Results:**

Between 2009 and 2017, 1,171,658 individual patients attended a health service participating in ACCESS network comprising 7,992,241 consultations. Regarding those with unique BBV and STI-related health needs, ACCESS captured data on 366,441 young heterosexuals, 96,985 gay and bisexual men, and 21,598 people living with HIV.

**Conclusions:**

ACCESS is a unique system with the ability to track efforts to control STIs and BBVs—including through the calculation of powerful epidemiological indicators—by identifying response gaps and facilitating the evaluation of programs and interventions. By anonymously linking patients between and within services and over time, ACCESS has exciting potential as a research and evaluation platform. Establishing a national health surveillance system requires close partnerships across the research, government, community, health, and technology sectors.

**International Registered Report Identifier (IRRID):**

DERR1-10.2196/11028

## Introduction

Globally, sexually transmissible infections (STIs) and blood-borne viruses (BBVs) are associated with significant morbidity, mortality, health costs, and social stigma. Indeed, these infections represent a major public health burden. There are, for example, nearly 37 million people currently infected with HIV and >1 million associated deaths per year [1], while an estimated 70 million people live with hepatitis C, from which nearly half a million die each year [2]. Regarding hepatitis B, >250 million people live with this infection, which causes >800,000 deaths annually [3,4]. Around the world, there are >350 million new cases of curable STIs every year—chlamydia, gonorrhea, syphilis, and trichomoniasis [5]—and human papillomavirus (HPV) is responsible for nearly all cases of cervical cancer, the fourth most common malignancy worldwide [6].

In Australia, the control and elimination of some BBVs and STIs are increasingly feasible through combinations of new and existing strategies of prevention, treatment, and management. For HIV, elimination is a tantalizing possibility through regular testing in combination with pre-exposure prophylaxis among uninfected individuals and antiretroviral treatment among those living with the virus [7-9]. Achieving something as lofty as HIV elimination will, naturally, be a major challenge [10] and certainly one that requires close monitoring of biomedical prevention coverage and impact to guide the refinement of implementation strategies [11]. Similarly, curative therapy with direct-acting antivirals for hepatitis C has been made available to all infected people in Australia, representing a major advance for both individual and public health [12] but one that also requires monitoring, evaluation, and adaptation if there is any hope of reducing infection rates [13].

For some other infections—notably hepatitis B and HPV— vaccinations have proved highly effective in reducing population incidence and prevalence. There remain, however, cohorts of people not included in vaccination schedules because of their age or who have migrated to Australia from countries where prevalence is high and vaccination programs limited. For these infections, ongoing clinical screening is required to identify unvaccinated individuals, and in the case of HPV, intervene early as a precursor to cancer. In addition, for curative STIs, frequent testing and timely treatment are fundamental components of interrupting incubation and preventing unintended onward transmission [14]. For STIs and BBVs, it is clear that ongoing efforts are required to track progress against targets, monitor population health, assess the impact of interventions, and plan into the future.

Surveillance and monitoring of BBVs and STIs are often complicated by the fact that they disproportionately affect populations defined by sexual identity, sex practice, drug use, and ethnicity [11,13,14]. Thus, their management requires a holistic and comprehensive approach to care, which in Australia and many other countries, involves sexual health clinics, targeted general practice clinics, drug and alcohol services, and hospitals. Health services like these play a vital role not only in diagnosing and managing BBVs and STIs but also in their prevention by encouraging uptake of diagnostic testing, treatment, and vaccines where available. Calculating the uptake of these initiatives, however, requires knowing the number of total attending patients—the denominator—and such information can only be sourced directly from health services. When linked between individuals’ episodes of care, these data can also be used to calculate other powerful impact indicators such as incidence or the time between diagnosis and treatment.

Here, we describe ACCESS—the Australian Collaboration for Coordinated Enhanced Sentinel Surveillance—a national system of sentinel surveillance that draws upon data from several different types of health services and pathology laboratories to inform and evaluate Australia’s BBV and STI control efforts.

## Methods

### Overview and Aims

ACCESS is a system that routinely extracts and collates line-listed, deidentified data from health services and pathology laboratories across Australia. Through anonymous patient linkage between and within services and laboratories, ACCESS produces a retrospective and prospective cohort of patients attending participating sites. Established in 2008, ACCESS began as a sentinel system for chlamydia surveillance [15] that was expanded in 2013 to include BBVs and other STIs in some Australian jurisdictions. Through funding from the Australian Department of Health in 2016, ACCESS expanded further to encompass a greater number and a more diverse selection of sites relevant to these infections in all 8 Australian states and territories.

The overall aim of ACCESS is to support the Australian response to STIs and BBVs by monitoring the testing, diagnosis, and management of these infections. In addition, ACCESS aims to operate as an evaluative platform to measure the impact and outcomes of relevant programs and interventions. This includes attention to Australian priority populations (gay and bisexual men and other men who have sex with men, people who use drugs, Aboriginal and Torres Strait Islander people, young heterosexuals, sex workers, and people from culturally and linguistically diverse backgrounds) and to “cascades of care” (eg, HIV) [16].

### Infections

ACCESS focuses on specific infections, including HIV, hepatitis B, hepatitis C, HPV, chlamydia, gonorrhea, syphilis, and trichomoniasis. The design of ACCESS, however, allows for the addition of other infections or conditions as required into the future. Already, for example, steps have been taken to begin collecting data on *Mycoplasma genitalium*, a newly identified STI.

### Sites and Recruitment

The ACCESS network seeks to include health services and pathology labs that best represent the prevention and management of BBVs and STIs nationally and in each state and territory. To be eligible, health services are required to use an electronic patient management system (ie, not based solely on paper files) and be willing to participate for a minimum of 2 years. Of note, we have encountered no health services still exclusively using paper files. In addition, health services have to see at least 50 individuals per year categorized as ≥1 of Australia’s priority populations for BBVs and STIs or they have to represent a service designed specifically for the care and management of these infections (eg, sexual health clinics and HIV testing sites). Given differences in the overall population size between each state and territory—the largest contains >7.5 million people, while the smallest has just >200,000—we are flexible in our assessment of caseloads to allow for recruitment in smaller jurisdictions. For example, while a caseload of 50 patients with HIV might be considered small or medium in New South Wales, it would be considered large, if not the largest, in Tasmania.

Within these parameters, per jurisdiction, we have sought to include a minimum of 2 sites with large caseloads of people living with or at risk of HIV, 2 with large caseloads of people living with or at risk of hepatitis and 2 with a large amount of STI-related testing and care. In addition, we sought to recruit 2 pathology laboratories per jurisdiction (one public and one private) that conducted testing for BBVs and STIs. While in larger states (New South Wales, Queensland, and Victoria), it was necessary to exceed these targets and recruit larger number of sites, the opposite was true in some smaller jurisdictions. In the Australian Capital Territory, for example, the vast majority of BBV-related and STI-related tests were conducted by a single pathology laboratory, making it unnecessary to recruit a second site of this kind. Details like this one highlight the need and strength for a tailored approach to recruitment, given the significant differences between each state and territory.

As noted, ACCESS has been in operation since 2008 with recruitment taking place over many years and in different iterations. Most recently, recruitment was undertaken from 2016 through 2018 to expand the network’s coverage in jurisdictions beyond New South Wales and Victoria. Over time, however, methods of identifying and recruiting sites have remained consistent; to gain jurisdictionally specific information on potential sites, we consult with local stakeholders in government, health, community organizations, and research institutes. We ask them to nominate sites that either conduct a large amount of testing for or care of BBVs and STIs, or sites that provide care for concentrations of Australia’s priority populations. Where available, we also review public health data on locations of BBV and STI diagnoses (by general geographic areas) to identify health services located nearby, and we review publicly available lists of doctors licensed to prescribe treatments for HIV and hepatitis C.

When this process was undertaken in 2016 and 2017, we identified 115 health services and 24 pathology laboratories across Australia that met our eligibility criteria. Three-quarters of these sites were already involved with ACCESS, noting that since its inception in 2008, no site has ever withdrawn participation in the network. Of the remaining “new” sites identified through our scoping process, we undertook purposive sampling that deferred to sites with large caseloads and those that introduced diversity either through their location, patients’ characteristics, or service model.

**Table 1:** Health services and pathology laboratories participating in the Australian Collaboration for Coordinated Enhanced Sentinel Surveillance network as of March 2018, by site type and jurisdiction.

Site type	Total	Jurisdiction							
		Australian Capital Territory	New South Wales	Northern Territory	Queensland	South Australia	Tasmania	Victoria	Western Australia
Sexual health clinic	58	1	38	3	8	1	3	2	2
General practice clinic	29	1	10	0	2	1	0	13	2
Hospital outpatient clinic	7	0	2	0	1	0	1	2	1
Community-led services	9	0	4	0	2	1	0	1	1
Drug and alcohol service	1	0	0	0	0	0	0	1	0
Private pathology laboratory	6	1	3	0	0	0	1	1	0
Public pathology laboratory	11	0	5	0	1	0	1	4	0

**Figure 1:**
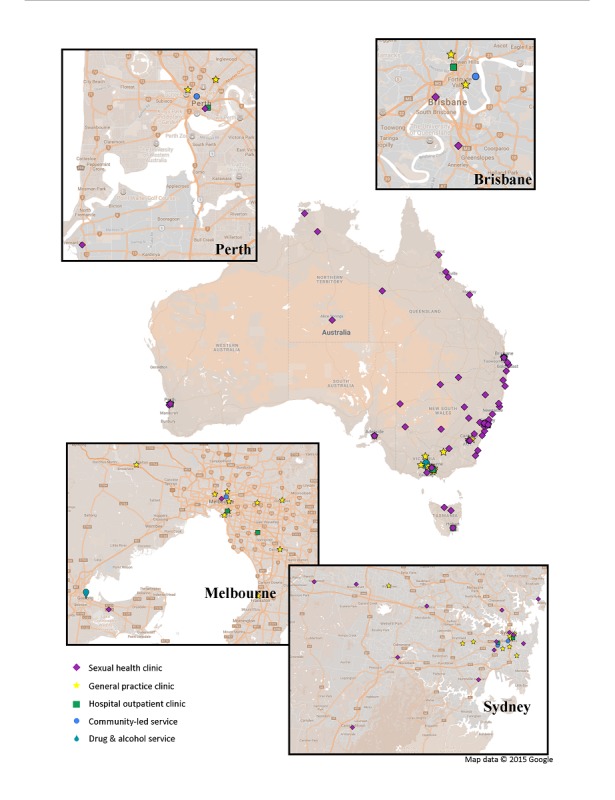
Health services participating in the Australian Collaboration for Coordinated Enhanced Sentinel Surveillance network as of March 2018.

A total of 31 new sites were recruited to ACCESS, resulting in 92% (104/113) of health service identified nationally and 71% (17/24) of laboratories identified nationally participating in ACCESS. Participation is being negotiated with further 11 sites, 3 refused participation altogether, and 4 were not pursued because of small patient caseloads compared with other similar services in their jurisdiction. Table 1 provides an overview of sites contributing data to ACCESS as of March 2018, noting that these numbers will continue to change over time. Figure 1 depicts participating sites on a map of Australia.

**Table 2:** Variables extracted (where available) from participating health services via Australian Collaboration for Coordinated Enhanced Sentinel Surveillance.

Domain	Variables
Patient information	Age Gender Home postcode Indigenous status Country of birth Preferred Language Year of arrival in Australia Sexual behavior
Consultation details	Date Type Reason for attendance Clinical diagnosis
Laboratory testing	Tests requested Results Specimen type Anatomical site
Treatment	Drug name Start or stop dates Dose Frequency Route
Vaccination details	Vaccination history Vaccines administered Type Dose number
Behavioral: sexual practices	Sexual partner gender(s) Sexual partner numbers Condom use Sex work Sex work location
Behavioral: drug use	Use of injecting drugs Shared injecting equipment Drugs injected

### Data Extraction and Management

Data are extracted from participating sites using software known as GRHANITE, which was designed specifically for the secure collection of deidentified health data. GRHANITE works by being installed on a local system at each participating site. Technical and financial costs associated with calibrating and installing GRHANITE are borne by ACCESS, making it a cost-neutral enterprise for sites. On an at least monthly basis, querying the site’s database or accessing files already extracted from the database is performed. The nature of these queries is guided by schemas customized to meet the requirements and structures of each database. Because of their flexibility, GRHANITE’s schemas can be deployed for use with almost any database structure to work across diverse systems ranging from established commercial platforms to unique systems built-for-purpose by individual services. Details of GRHANITE have been published previously [17,18]. Table 2 lists variables extracted for ACCESS, where available. Some sites routinely collect behavioral information from patients, and where available, these data are also extracted.

As part of the extraction process, GRHANITE removes any data that could potentially identify a patient. While still working within a participating site, GRHANITE generates probabilistic record linkage keys (or signatures) called “hashes.” These signatures are derived from, but do not contain, personal information, which means that probabilistic linkage can be safely conducted between and within participating ACCESS sites. This process makes it possible to monitor patient movement between services in a way that is anonymous and ensures that no identifiable patient details are ever transmitted beyond participating sites. Following extraction, GRHANITE encrypts the data and transmits it to a central, secure server.

Data are extracted for all patients and consultations, but pathology and treatment data are only extracted when there is evidence of testing or treatment for an STI or BBV. This approach helps limit the size of the ACCESS database by focusing on the most relevant pathology testing. In general practice clinics, notably, the majority of testing would be unrelated to these infections, which would place an unnecessarily high technical burden on systems to fully extract all data. Thus, filters are used and regularly reviewed to ensure accuracy and completeness.

ACCESS data are processed using code that organizes pathology testing, treatment, and patient details into standardized formats. This step also involves unifying the structure of data received from different systems. Notably, because many patient management systems store pathology results as free text, computational parsing is used to identify test names, dates, and results to organize that information into consistent categories. This code is regularly reviewed for accuracy and adapted over time, as required. A similar process is used to identify patients that form part of Australia’s priority populations, which involves drawing upon numerous pieces of information (eg, demographics, behavioral details, and pathology tests) to properly categorize patient files. For example, previous research has found that sexual orientation is not well recorded among men attending Australian general practice clinics [19]. To address this issue, we use history of rectal swabs for STI testing as a proximal marker indicating anal sex, which has previously been found to be effective for identifying gay and bisexual men and other men who have sex with men [20]. Definitions for organizing pathology tests and categorizing patients into priority populations were constructed in close consultation with relevant clinical and laboratory experts, as well as community representatives.

### Data Quality

The quality of ACCESS data is ensured through a number of processes. Data extracted from new services are validated through a consultative process with site investigators, which includes sharing preliminary outputs to gauge the degree to which they converge (or diverge) from clinical experience. This feedback is then used to improve data processing and address gaps or errors in the extraction process. For example, to ensure data completeness, we might ask clinic staff to estimate the number of HIV-positive patients they see each year or the number of chlamydia tests they conduct in an average week, which can then be compared against extracted and processed data. This process has previously identified components missing from extracts, including pathology test names, drugs types, and demographic variables, and then used to adapt and correct extraction processes.

Beyond completeness, we also carefully attend to the accuracy of ACCESS data. This involves what is extracted as well as how we process extracted data, which is to say how variables are organized into distinct and consistent categories. Wherever possible, ACCESS data are triangulated with other sources to improve accuracy. This process includes comparing extracted health service data to that from pathology laboratories; because some participating laboratories serve participating health services, we can assess the degree to which the number of tests and results align. Comparing data in this way has allowed us to refine pathology filters and our processes for organizing results. In the past, ACCESS data have also been assessed for accuracy against passive surveillance information. For example, we previously requested information on HIV and STI notifications among sexual health clinic attendees in New South Wales to calibrate our systems for processing diagnoses in these clinics [21].

Routine data quality checks are also conducted on a quarterly basis, which focus on assessing if there are significant changes in test frequencies over time to generate alerts for significant deviations. For example, if the number of tests extracted for chlamydia doubled from one period to the next, this would be used as a point of investigation. Investigations include reviewing data processing, checking raw data, and consulting with site investigators. This kind of quality assurance is done on the dataset as a whole, by health service type and to the level of individual sites.

### Dissemination and Use

ACCESS data are used for diverse purposes. Data extracted via ACCESS can be used to generate a number of powerful indicators relevant to BBVs and STIs, most commonly those related to diagnostic testing (test uptake, test frequency, test comprehensiveness, and retesting), treatment (treatment uptake and treatment success), infections (test yield, test positivity, incidence, and reinfection), and vaccinations (coverage of vaccine-acquired immunity). Indeed, indicators like these form part of ACCESS’s contributions to the national surveillance of BBVs and STIs [22] and their surveillance by individual states and territories, including as stand-alone reports or as part of existing reporting mechanisms [23]. In reports such as these, ACCESS has been used to improve estimates of treatment uptake and success, which supports more accurate “cascades of care” for HIV and other infections. The automated nature of data extraction and processing facilitates timely production of reports, which in some cases are published as early as 4 weeks from the end of a reporting period. Furthermore, site-specific ACCESS data are routinely reported back to participating sites, which can include analyses of testing uptake, test positivity, and diagnosis frequency.

In addition to routine surveillance reporting, ACCESS data are used for a number of other related projects. Notably, ACCESS data have been used in stand-alone analyses of population health, for example, in studies of HIV and STIs among sex workers in Australia [24,25] and an analysis of hepatitis C testing and diagnoses among people living with HIV [26]. Moreover, ACCESS data have been used to assess the impact of syphilis testing interventions [27]. Beyond this work, ACCESS is being used increasingly to support other forms of research and evaluation. In some projects, ACCESS provides line-listed and deidentified datasets, which are and have been used to conduct a large-scale study of HIV treatment-as-prevention [28], evaluate pre-exposure prophylaxis implementation trials [29,30], and study Victoria’s hepatitis C elimination response [31]. In other cases, as in the evaluation of HIV control in New South Wales [32], ACCESS has routinely provided specifically designed indicators (eg, HIV testing uptake and rates of viral suppression) to monitor and evaluate various aspects of BBV prevention and management. In many of these examples, ACCESS fills an important role by providing the kinds of data and indicators that are required for research of this kind to be conducted. Through these projects, ACCESS demonstrates its capacity to support diverse research on STIs and BBVs, which extends beyond the realms of surveillance and monitoring.

### Ethics and Governance

Ethical approval was granted by the lead human research ethics committee of Alfred Hospital in Melbourne (248/17), University of Tasmania (H0010220), and the Menzies School of Health Research (08/047). All ethics committees waived the need for consent to be collected from individual patients. Furthermore, ethical reviews were provided by organizations representing key populations, notably gay and bisexual men, people living with HIV, sex workers, and Aboriginal and Torres Strait Islander people.

To protect the identities of individual patients, access to the line-listed database is restricted to a small and select group of researchers. Where data are shared with others, potentially identifying details (eg, patient postcode) are replaced with broad categories (eg, urban/nonurban), which is a similar approach taken in any reporting of ACCESS data. Furthermore, analyses that produce cell counts of <5 individuals are suppressed.

An advisory committee was established comprising representatives from government organizations, community groups, health services and laboratories, and research institutes. This committee provides advice on the project’s development and conduct; promotes its aims and objectives; and contributes to analysis, interpretation, and dissemination.

## Results

Although some sites were able to provide electronic data going back as far as the 1980s, data quality and completeness tends to diminish further back in time when health services were less familiar with technologies of electronic health that dominate today. To examine a more recent period, we note that ACCESS captured data from a total of 1,171,658 individual patients who attended a participating health service at least once in the recent past between 1 January 2009 and 31 December 2017. These patients attended for a total of 7,992,241 clinical consultations or an average of 0.8 consultations per patient per year. Patient gender was evenly represented between men (597,545/ 1,171,658, 51%) and women (574,112/1,171,658, 49%), and records were extracted from a total of 1116 transgender patients (380/1116, 34% transgender men; 356/1116, 32% transgender women, and 380/1116, 34% unspecified gender).

Specific to Australia’s priority populations, from 2009 to 2017, ACCESS captured data of 366,441 young heterosexuals aged 16-29 years. In addition, the network includes data from 96,985 gay and bisexual men and other men who have sex with men. Data were also captured from 21,598 people living with HIV, drawing upon recorded HIV diagnoses, confirmed HIV pathology results, viral load testing, and clinical attendance for “HIV management.” In total, 22,089 Aboriginal and Torres Strait Islander patients attended an ACCESS health service during this period, noting that this variable was incomplete for 74% (576,219/778,674) of patients attending general practice clinics and 50% (196,490/392,984) of patients attending other services. Even though Australian guidelines recommend collecting indigenous status from all patients [33], it seems that this indicator is still not routinely collected.

As noted, sexual health clinics in Australia routinely collect enhanced behavioral data on factors associated with BBVs and STIs. This information is used by ACCESS to further identify members of priority populations. From 2009 to 2017, for example, it is possible to identify 12,111 people who attended an ACCESS site and reported injecting drug use at least once in the 12 months prior to consultation, as it is possible to identify 21,205 men and women who reported sex work in the previous 12 months. As noted, identifying members of these priority populations is not possible in settings other than sexual health and community-lead clinics, which is attributed to a lack of standardized methods for collecting and recording behavioral data. Work is ongoing to support the implementation of behavioral surveys in some general practice clinics and to develop algorithms for recognizing these populations through other means, such as through certain types and patterns of pathology tests and testing.

## Discussion

In this paper, we described the methods used to establish a national sentinel surveillance system for BBVs and STIs. ACCESS seeks to complement the existing passive surveillance by tracking the uptake and impact of strategies aimed at controlling these infections. The system is highly flexible and can be adapted for use in a multitude of health contexts and evolve over time to address emerging surveillance needs. In addition, it is a project deeply rooted in collaboration, involving government, researchers, community, and clinicians from every corner of Australia. ACCESS is a unique national resource and a model with potential relevance for other countries.

A key strength of ACCESS is its ability to anonymously link patients between services and over time. In some ways, this feature makes ACCESS akin to a national retrospective and prospective cohort, which has exciting possibilities in a number of areas. ACCESS allows scrutiny of the ways that individuals move through different pathways of care, including the overall trajectory and the time it takes to move from diagnosis to viral suppression or cure. Furthermore, this linkage facilitates the calculation of powerful epidemiological markers, like incidence and test frequency and also allows for examinations of compliance with clinical guidelines associated with testing (eg, chlamydia testing among young people presenting to clinics or following past positive tests). ACCESS also makes possible detailed, individual, and large-scale evaluations of public health policy, interventions, and other strategies aimed at controlling BBVs and STIs.

Another key strength of ACCESS relates to its coverage. Specifically, the network of health services in every state and territory enables comparison between not only Australian jurisdictions but also different types of service models, such as community-based testing services, sexual health clinics, and hospitals. These comparisons are important for identifying gaps, comparing the utility of different ways for providing care and nuanced information on how BBVs and STIs are diagnosed and managed. Furthermore, by attending to the geographic concentrations of Australia’s priority populations and working with community groups and health experts, ACCESS has collated some of the largest samples of “high-risk” priority populations seen anywhere in the world.

The automated nature of ACCESS significantly reduces the resources and time required to report surveillance data, benefits that are already being realized through quarterly reporting to state health departments. Although initial enrollment of new sites to ACCESS requires some time, maintenance is minimal once established, which helps ensure the system’s ongoing sustainability. Moreover, participating sites realize benefits through the publication of scientific research and the ability to more readily access their own data, including through tailored site reports that can include comparisons with aggregated data from similar sites. These strengths are reflected in the observation that in a decade of operation, no site has yet chosen to withdraw from ACCESS.

There are some limitations of the system that warrant consideration. As a surveillance network, ACCESS does not capture all new diagnoses and is, therefore, not a replacement for passive surveillance. Although we have described the process for anonymously linking patients between ACCESS sites, gaps arise when patients attend health services outside of the network. These gaps can be partly overcome through data from participating laboratories but they are inherent in the network’s “sentinel” nature. Another limitation is ACCESS’s inability to collect all clinical information, in particular, the free text detail contained within patient notes. Patient notes contain a wealth of details that would likely be relevant to BBVs and STIs but are not accessed by this system because they can potentially contain identifying information. Options for identifying and extracting relevant details through the use of text-recognition software are currently being assessed as a potential means of using this information confidentially. Finally, ACCESS is entirely reliant on routinely recorded health information; the quality and completeness of these details can vary between and within sites. This limitation, however, can be overcome in some cases with ACCESS’s capacity for anonymous patient linkage by pooling information from multiple services and laboratories to construct a more complete picture.

ACCESS represents a new way of conducting sentinel surveillance, which adds value for government, research, clinical, and community partners. With data extraction under way across the country, over the coming years, the project will focus on new ways of providing regular feedback to health service and laboratory sites as a way to improve service delivery, sustain interest, and capitalize on the network’s potential. In the future, it is imagined that ACCESS will continue to develop as a readily accessible resource for diverse stakeholders that seek to make use of it as a unique, national database.

## Abbreviations

ACCESS: Australian Collaboration for Coordinated Enhanced Sentinel Surveillance
BBV: blood-borne virus
HPV: human papillomavirus
STI: sexually transmissible infection
